# Case report: Rapid symptom relief in autoimmune encephalitis with efgartigimod: a three-patient case series

**DOI:** 10.3389/fimmu.2024.1444288

**Published:** 2024-10-03

**Authors:** Qianqian Zhang, Wenping Yang, Yun Qian, Yu Zhang, Huihui Zhao, Mingzhu Shu, Qingyang Li, Yanan Li, Yu Ding, Shiyu Shi, Yaxi Liu, Xi Cheng, Qi Niu

**Affiliations:** Department of Geriatrics, The First Affiliated Hospital of Nanjing Medical University, Nanjing Medical University, Nanjing, China

**Keywords:** autoimmune encephalitis, efgartigimod, GABABR, Lgi1, NMDAR, neonatal Fc receptor

## Abstract

**Introduction:**

Autoimmune encephalitis (AE) comprises a group of inflammatory brain disorders mediated by autoimmune responses. Anti–N-methyl-D-aspartate receptor (NMDAR) encephalitis, anti–leucine-rich glioma-inactivated 1 (LGI1) encephalitis, and anti–γ-aminobutyric acid-B receptor (GABABR) encephalitis are the most prevalent forms, characterized by the presence of antibodies against neuronal cell-surface antigens. Efgartigimod, an antagonist of the neonatal Fc receptor, has proven efficacy in myasthenia gravis treatment. This clinical case report describes the clinical progression and functional outcomes of AE in three patients who received efgartigimod treatment.

**Case presentations:**

Case 1 was a 60-year-old man exhibiting memory impairment and psychiatric disturbances over 11 days. Case 2 was a 38-year-old man with a 1-month history of rapid cognitive decline and seizures. Case 3 was a 68-year-old woman with mental behavioral changes and seizures for 4 months. Anti-GABABR, anti-LGI1, and anti-NMDAR antibodies were confirmed in the respective patients’ cerebrospinal fluid or serum. All three patients experienced marked and swift symptomatic relief after four cycles of efgartigimod treatment, with no complication.

**Conclusion:**

Current first-line and second-line treatments for AE have limitations, and efgartigimod has demonstrated potential in the rapid and efficacious treatment of AE, emerging as a promising option for the management of this disease.

## Introduction

Anti–N-methyl-D-aspartate receptor (NMDAR) encephalitis is the most common form of autoimmune encephalitis (AE), accounting for approximately 54–80% of AE cases (1–3). It is followed by anti–leucine-rich glioma-inactivated 1 (LGI1) encephalitis and anti–γ-aminobutyric acid-B receptor (GABABR) encephalitis. These three types of encephalitis involve the actions of antibodies against neuronal cell-surface antigens and cause neuronal dysfunction primarily through humoral immune mechanisms (1–3). The immunoglobulin (IgG) subtypes associated with these different phenotypes differ; anti-LGI1 antibodies are associated predominantly with IgG4, whereas anti-GABABR and NMDAR antibodies are associated primarily with IgG1 (4, 5). AE typically presents with psychiatric and behavioral abnormalities, cognitive impairment, recent memory decline, and seizures, with clinical manifestations varying depending on the mediated antibody (6).

Apart from the symptomatic management of seizures and psychiatric symptoms, the current treatment of AE is predominantly immunosuppressive, involving an escalating approach. First-line immunotherapy, initiated during the acute phase of the disease, involves primarily the administration of high-dose corticosteroids and intravenous immunoglobulin (IVIG), or plasmapheresis. When it is ineffective, second-line treatments such as rituximab or cyclophosphamide administration are considered (7). As an invasive procedure, plasmapheresis increases the risk of infection, can induce coagulopathy, and is especially challenging to administer to uncooperative patients and children (8, 9). IVIG is not only costly and dependent on plasma donations, but also increases the lysosomal degradation of normal Igs while enhancing the degradation of pathogenic ones, and some patients respond poorly to it (10). Moreover, although some observational studies have suggested that second-line immunotherapy correlates with improved functional outcomes (4, 5, 9, 11), recent meta-analyses have not supported the association of second-line drug administration with such improvement in AE subgroups (12). Importantly, 19–33% of patients with AE do not respond well to first- or second-line treatments and continue to have persistent neurological and psychological issues (4, 6, 7, 11, 13, 14). Thus, effective, targeted, and well-tolerated treatments for AE are urgently needed.

Neonatal Fc receptors (FcRns), located primarily in the reticuloendothelial system, regulate the intracellular and transcellular transport of IgG, protecting it (and pathogenic antibodies) from degradation by intracellular lysosomes and maintaining its extended half-life (15). FcRn antagonists work by specifically blocking the binding of FcRn to IgG, thereby accelerating its degradation, and they do not significantly affect other immunoglobulin subclasses, such as IgA and IgM (16). Efgartigimod, as the first FcRn antagonist, has been approved for the treatment of myasthenia gravis (MG) in the United States, Europe, Japan (8, 17), and China (https://www.nmpa.gov.cn/zwfw/sdxx/sdxxyp/yppjfb/20230704155106142.html). It has also shown tremendous therapeutic potential for other autoimmune diseases, such as primary immune thrombocytopenia and pemphigus vulgaris/pemphigus foliaceus (9, 16).

Reports on the use of FcRn antagonists for the treatment of AE are currently lacking. Here, we present three cases of AE (one each of anti–LGI1 antibody encephalitis, anti-GABABR encephalitis, and anti–NMDAR antibody encephalitis) that were treated with four cycles of efgartigimod. This reporting has been approved by the institutional ethics committee of the First Affiliated Hospital of Nanjing Medical University (2024-SR-458), and is in line with the 2020 Surgical CAse REport criteria (18).

## Case presentations

### Patient 1

**Figure 1:**
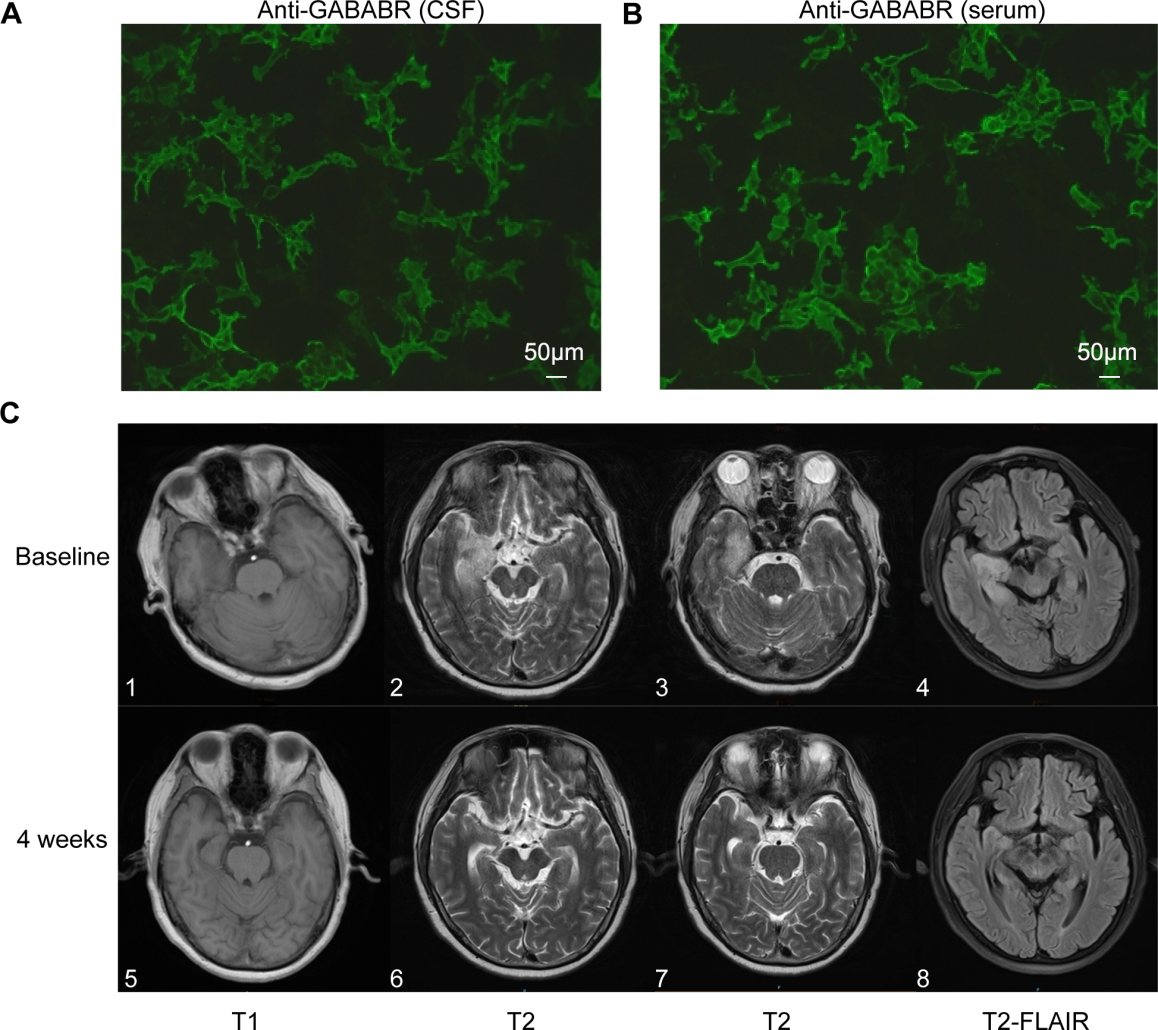
GABABR-transfected cells and anterior and posterior changes in cranial MRI features of patient 1. **(A, B)** show CSF and serum positivity for the GABABR antibody, respectively (Bar: 50 μm). **(C)** 1-4 shows lesions with abnormal signals in the right temporal lobe and hippocampus on T1, T2, and FLAIR sequences prior to efgartigimod treatment. **(C)** 5-8 shows the reduction of lesion signals and narrowing of the range after 4 weeks of efgartigimod treatment. GABABR, γ-aminobutyric acid-B receptor; MRI, magnetic resonance imaging; CSF, cerebrospinal fluid; FLAIR, fluid attenuated inversion recovery.

**Figure 2:**
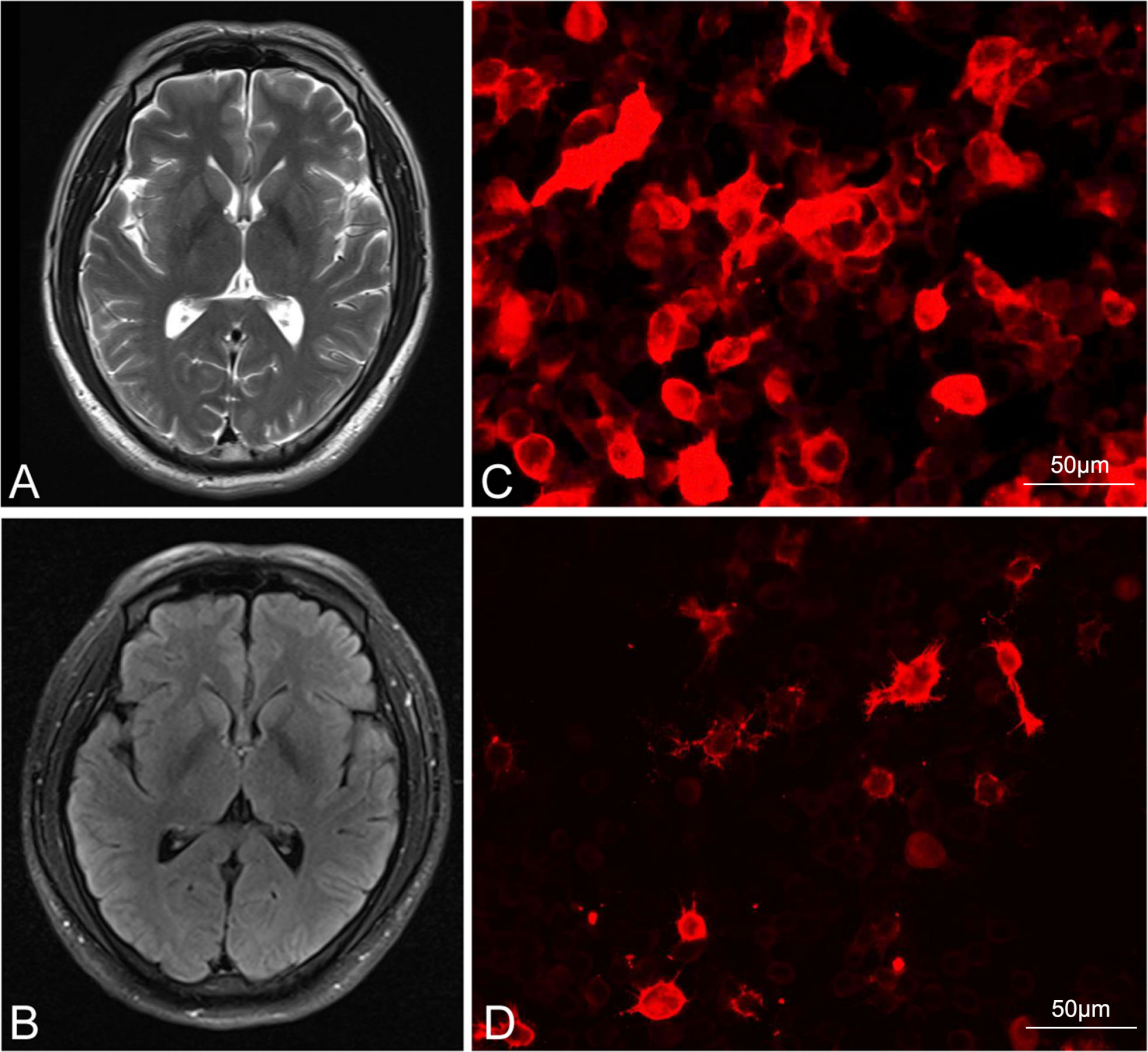
Brain imaging and serum anti-LGI1 results for patient 2, and serum anti-NMDAR results for patient 3. **(A, B)** show the lack of abnormal signaling on MRI for patient 2. **(C, D)** show serum positivity for the LGI1 antibody and anti-NMDAR in patients 2 and 3, respectively (Bar: 50 μm). LGI1, leucine-rich glioma-inactivated 1; NMDAR, N-methyl-D-aspartate receptor; MRI, magnetic resonance imaging.

The first patient was a 60-year-old Chinese man with a 20-year history of smoking and alcohol consumption, with no other chronic disease, specific infectious disease, or familial genetic disorder. On the day of symptom onset, he was brought to a local hospital by family member due to abnormal behavior, sluggish responses, memory decline, and altered mental status. An initial physical examination revealed cognitive decline, including a lack of concentration, spatiotemporal disorientation, impaired working memory, and loss of abstract thinking ability. Initial imaging studies yielded no specific finding, and evoked potentials indicated cognitive function impairment. Laboratory tests ruled out specific infections (e.g., HIV and syphilis), tumors, and metabolic diseases. Cerebrospinal fluid (CSF) analysis showed increases in white blood cells and total IgG, and antibody component analysis confirmed CSF and serum anti-GABABR antibody IgG positivity [1:10 (++) and 1:100 (++), respectively; **Figure 1A, B**]. On the sixth day, the patient was admitted to the local hospital for 5 days of immunomodulatory treatment with methylprednisolone (1000 mg/day) and IVIG (25 g/day), but his symptoms did not improve significantly. On the 11^th^ day after symptom onset, he was transferred to our department for further treatment. Repeat cranial magnetic resonance imaging (MRI) showed abnormal patchy signals in the patient’s right temporal lobe and hippocampus (**Figure 1C**). Chest and abdominal computed tomography (CT) examination revealed no sign of a tumor. An electroencephalogram showed slow spike-wave complexes in the right temporal region. Based on the diagnostic criteria for AE (19), the patient was diagnosed with anti-GABABR encephalitis. During the first week, we continued with gradually tapered steroid therapy combined with an antiepileptic agent, nutritional support, and other symptomatic treatments, but with no significant effect (**Table 1**). On the 18^th^ day, we initiated intravenous injections of efgartigimod (800 mg once weekly) in conjunction with intravenous methylprednisolone (80 mg/day). After two efgartigimod infusions, the patient demonstrated clinical improvement. On the 32^nd^ day, we discontinued the methylprednisolone and transitioned the patient to oral prednisone acetate (50 mg/day), with 5 mg tapering every 2 weeks. On the 46^th^ day, after completing four cycles of efgartigimod treatment, the patient’s psychiatric symptoms had disappeared, his cognition had returned to normal, and no adverse event had occurred. The Clinical Assessment Scale in Autoimmune Encephalitis (CASE) (20) and other scores showed significant improvements (**Table 1**, **Figure 2**). Follow-up cranial MRI showed significant reduction of the abnormal signals in the right temporal lobe and hippocampus (**Figure 1C**). After 8 weeks of treatment with efgartigimod, we observed a substantial reduction in the serum anti-GABABR antibody titer to 1:32.

**Table 1:** Clinical data of patients with AE treated with efgartigimod.

Characteristic	Patient 1	Patient 2	Patient 3
Age (years)	60	38	68
Sex	M	M	F
Education (years)	19	16	9
Age at AE onset (years)	60	38	67
AE subtype	Anti-GABABR	Anti-LGI1	Anti-NMDAR
Personal history	Smoking and alcohol consumption	Smoking and alcohol consumption	None
Basic treatment	IVIG and cortisol	Cortisol	IVIG and cortisol
MMSE score			
Baseline	19	13	11
1 week	22	24	17
2 weeks	24	27	22
4 weeks	27	27	24
CAM score			
Baseline	30	29	26
1 week	22	17	19
2 weeks	20	17	13
4 weeks	16	9	11
mRS score			
Baseline	4	3	3
1 week	3	2	3
2 weeks	3	2	2
4 weeks	2	1	1
CASE score			
Baseline	9	12	11
1 week	6	4	7
2 weeks	5	3	6
4 weeks	2	2	3

AE, autoimmune encephalitis; GABABR, γ-aminobutyric acid-B receptor; LGI1, leucine-rich glioma-inactivated 1; NMDAR, N-methyl-D-aspartate receptor; IVIG, intravenous immunoglobulin; MMSE, Mini-Mental State Examination; CAM, confusion assessment method; mRS, Modified Rankin Scale; CASE, Clinical Assessment Scale in Autoimmune Encephalitis.

### Patient 2

**Figure 3:**
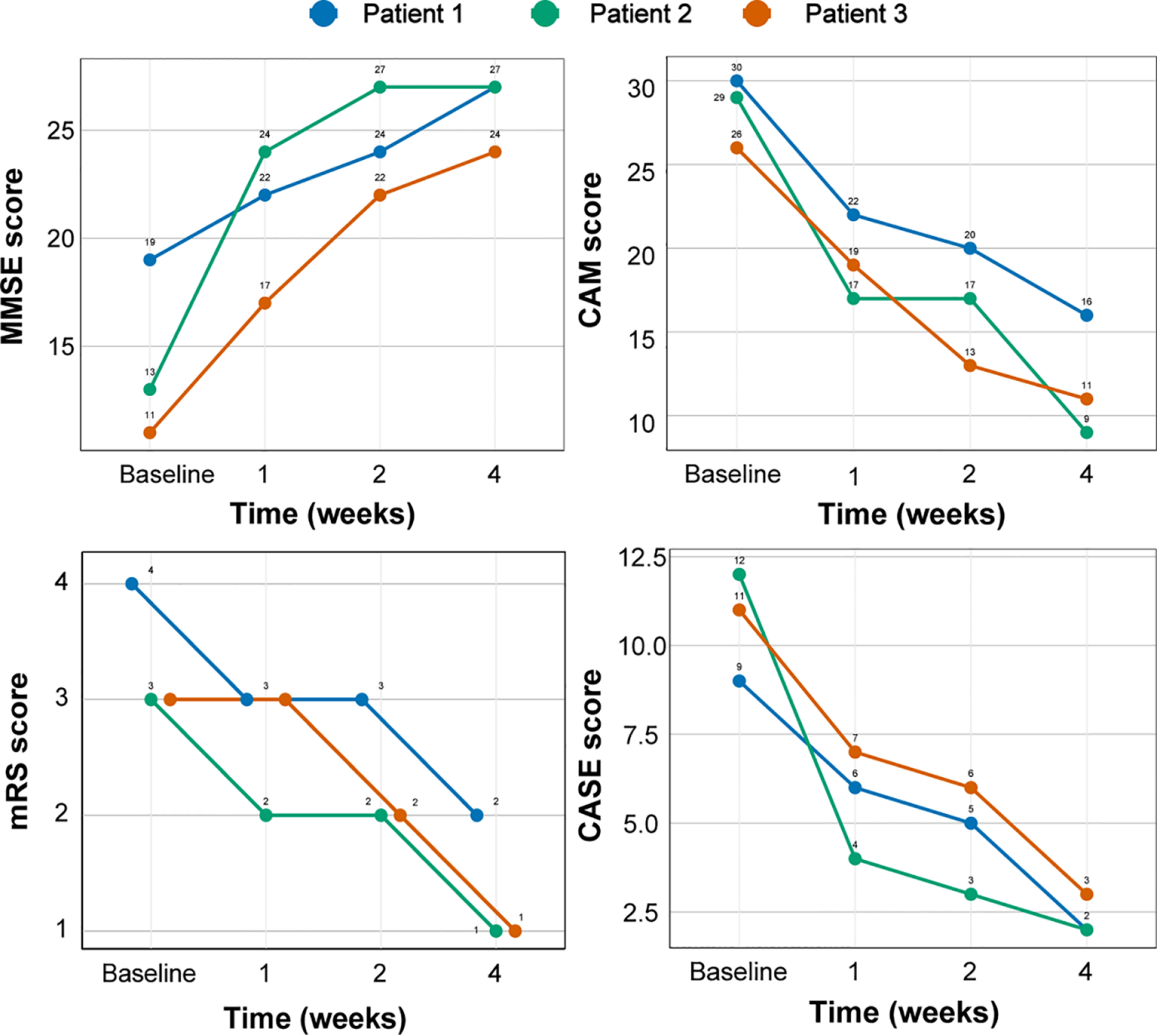
Changes in the clinical scores of three patients with AE with efgartigimod treatment. AE, autoimmune encephalitis; MMSE, Mini-Mental State Examination; CAM, confusion assessment method; mRS, Modified Rankin Scale; CASE, Clinical Assessment Scale in Autoimmune Encephalitis.

The second patient was a 38-year-old Chinese man with a 20-year history of smoking and alcohol consumption but no other significant medical or medication history. He sought medical attention at a local hospital 30 days after experiencing three episodes of limb convulsions, accompanied by memory decline and impaired speech. Cranial MRI showed no abnormality, and the patient was provisionally diagnosed with epilepsy and prescribed oral levetiracetam (**Supplementary Figure S1**). However, his symptoms intensified progressively, accompanied by the emergence of hallucinations, psychiatric symptoms, and episodes of nocturnal sleep disruption. On the 45^th^ day, the patient was referred to our hospital. Initial physical examination indicated cognitive decline, sluggish responses, and disorganized speech. Repeat cranial MRI (**Figures 3A, B**) and chest CT examinations revealed no specific finding. Laboratory tests showed only a slight decrease in the serum sodium concentration (136.5 mmol/L). Although we were unable to obtain CSF due to the family’s refusal of lumbar puncture, serum analysis revealed anti-LGI1 IgG antibody positivity at a titer of 1:100 (**Figure 3C**). Based on the AE diagnostic criteria (10), the patient was definitively diagnosed with anti-LGI1 encephalitis. On the 52^nd^ day after symptom onset, we commenced treatment with intravenous sodium efgartigimod (800 mg weekly). As the patient’s family declined high-dose steroid therapy, a combined treatment approach with methylprednisolone (80 mg/day) and a planned tapering regimen was adopted. On the 59^th^ day, substantial improvement of all symptoms was observed: the patient’s seizures had ceased, his memory and speech capabilities had improved, his hallucinations had disappeared, and his daily living abilities had increased. On the 80^th^ day, after the receipt of four efgartigimod infusions, improvement in all evaluation scale scores was noted and no adverse event had occurred (**Table 1**, **Figure 2**), On the 108^th^ day, a repeat serum anti-LGI1 antibody IgG titer demonstrated a decrease to 1:10.

### Patient 3

The third patient was a 68-year-old previously healthy Chinese woman with no notable medical or medication history. On the first day of symptom onset, she received intravenous fluids and antibiotics due to a low-grade fever at a local clinic. On the seventh day, she developed confusion and involuntary limb convulsions and was admitted to the intensive care unit of a local hospital. Investigations, including cranial MRI, echocardiography, and electrocardiography, showed no abnormality (**Supplementary Figure S1**). The patient gradually developed memory decline, speech impairment, auditory and visual hallucinations, agitation, and aggressive behavior. On the 10^th^ day, the local hospital suspected viral encephalitis with possible AE and treated her with corticosteroids and IVIG for 5 days, after which her seizures did not recur. On the 17^th^ day, the patient was discharged with a tapering oral corticosteroid regimen. However, she continued to experience cognitive impairment and psychiatric disturbances, developing paranoid delusions and nocturnal delirium that rendered her incapable of caring for herself. On the 125^th^ day after symptom onset, the patient was brought to our outpatient clinic, where her initial CASE score was determined to be 11 (**Table 1**). Laboratory test findings were unremarkable, but serum antibody analysis revealed anti-NMDAR antibody IgG positivity [1:10 (+); **Figure 3D**]. According to the AE diagnostic criteria (19), the patient was definitively diagnosed with anti-NMDAR AE. On the 132^nd^ day after symptom onset, she was prescribed oral prednisone acetate (40 mg once daily) and intravenous efgartigimod (800 mg weekly). After 2 weeks of treatment, significant improvements (reduction of nocturnal delirium; disappearance of hallucinations, agitation, and aggression; and partial independence in daily living activities) were noted. On the 160^th^ day, after 4 weeks of efgartigimod treatment, the patient was mostly independent in daily living activities, with no occurrence of leukopenia, headache, or respiratory or urinary infection, and showed continued improvement on her follow-up scale assessments (**Figure 2**). Post-treatment antibody titer data for patient 3 were unobtainable due to her family’s refusal of further testing.

## Discussion

Despite the presence of different antibody targets, all three AE cases described in this report exhibited rapid and significant clinical improvement following efgartigimod treatment. Importantly, we observed no adverse event such as respiratory or urinary tract infection or headache, reported to be the most common adverse reactions (19). Significantly, our three patients commenced efgartigimod treatment at 18, 52, and 132 days after symptom onset, corresponding roughly to the early, middle, and late stages of AE, respectively. All patients exhibited rapid primary symptom improvement, regardless of prior adequate conventional immunotherapy exposure. Although potential synergistic or additive effects of standard therapies cannot be excluded definitively, our cases suggest that efgartigimod treatment is appropriate across various AE stages, and thus that the medication demonstrates promising therapeutic potential. Furthermore, converging lines of evidence suggest that efgartigimod is effective across the spectrum of NSAb-associated AE subtypes. This possibility is supported by compelling preclinical data (20), the high prevalence of anti-LGI1 and anti-NMDAR encephalitis subtypes within this spectrum (21), and recently published findings confirming the positive effects of efgartigimod on AE (22, 23). The consistent improvement observed in our case series, which encompasses the three most common NSAb-associated AE subtypes, further strengthens this notion. Given that efgartigimod reduces all IgG subclasses (24) and AE is mediated primarily by IgG1 and IgG4 antibodies (4, 5), this medication shows promise for broad application, although further investigation is required.

Anti-GABABR encephalitis is diagnosed in 5.6% of patients with AE (3). Early MRI findings are often normal in these patients, and findings may remain negative even after symptoms involving the limbic system appear (11, 25). This presentation aligns with the case of patient 1, whose initial imaging examination showed no abnormality. Although anti-GABABR encephalitis is associated with small cell lung cancer in approximately one-third of patients (26), two chest CT examinations and tumor marker screening revealed no sign of a tumor in patient 1. The prognosis for non-paraneoplastic anti-GABABR encephalitis is better than that for tumor-associated disease (12, 27), and proactive immunotherapy can significantly improve neurological outcomes in patients with anti-GABABR encephalitis (28). Although initial treatment with high-dose steroids and IVIG did not significantly improve patient 1’s symptoms, marked improvement was observed after two injections of efgartigimod. By the end of the 4-week treatment period, all of this patient’s assessment scale scores were close to normal, confirming the significant therapeutic effects of efgartigimod and its potential as a treatment option for anti-GABABR encephalitis.

Anti-LGI1 encephalitis, first reported in 2010 (29), is the second most common type of AE after anti-NMDAR encephalitis. Its primary clinical manifestations are acute or subacute cognitive impairment, psychiatric and behavioral disturbances, sleep disorders, faciobrachial dystonic seizures, epileptic seizures, and refractory hyponatremia (30). Patient 2, who was relatively young, had a slightly reduced serum sodium level but no sign of a concurrent tumor. Most cases of anti-LGI1 encephalitis are non-paraneoplastic, with good prognoses and low recurrence rates; outcomes are worse for patients with hyponatremia, older age, poor initial treatment response, and recurrent disease (31, 32). Thus, early diagnosis and more aggressive immunotherapy may contribute to prognosis improvement (33). Patient 2 showed significant symptom improvement within 1 week of the initiation of treatment with low-dose corticosteroids and efgartigimod, with no notable adverse event. Two large real-world studies conducted in the United States and Italy demonstrated that efgartigimod effectively reduces glucocorticoid requirements during maintenance therapy for MG (34, 35). The treatment course of patient 2 suggests that efgartigimod similarly reduces corticosteroid dosage needs for AE, potentially mitigating the occurrence of side effects and improving treatment adherence. Our findings are consistent with those presented in two recently published articles (22, 23) and further confirm the therapeutic benefits of efgartigimod for LGI1-antibody encephalitis.

Anti-NMDAR encephalitis is the most common subtype of AE, typically presenting in adults with acute behavioral changes, psychosis, seizures, memory decline, motor disturbances, and autonomic dysfunction (2, 36). In this context, Glasgow Coma Scale scores ≤ 8 at admission, cognitive impairment, serum antibody positivity, and the delay of immunotherapy are risk factors for poor prognosis at discharge, whereas early immunotherapy initiation may improve outcomes (37, 38). However, up to one-third of patients with AE do not respond to standard immunotherapy (39). Patient 3 experienced no improvement in her psychiatric symptoms despite the prompt initiation of corticosteroid and IVIG therapy, which may have contributed to the low antibody titer detected after 4 months. Her symptoms had improved after 2 weeks of efgartigimod treatment, suggesting that Fc receptor antagonists are an effective alternative when standard immunotherapy is ineffective, although further clinical cohort studies are needed to confirm this possibility.

The potential role of efgartigimod in the treatment of patients with AE is an intriguing avenue for further investigation. As a large protein molecule with a molecular weight of 54,000 Da (40), efgartigimod’s capacity to passively diffuse across an intact blood–brain barrier (BBB) is theoretically limited. However, FcRn, a receptor that is highly expressed on cerebral microvascular endothelium and choroid plexus epithelium, plays a pivotal role in IgG transport across the BBB (10). Whereas FcRn typically mediates IgG efflux from the brain into the circulation under physiological conditions, it can facilitate reverse transport – the influx of IgG antibodies from the bloodstream into the central nervous system (CNS) – under conditions of inflammatory or BBB compromise (10, 41). Animal studies have demonstrated that anti-FcRn antibodies reduce disease activity by inducing the degradation of disease-specific antibodies in the brain and spinal cord (20). Given the well-documented increased in BBB permeability often observed in inflammatory autoimmune CNS diseases, such as myelin oligodendrocyte glycoprotein antibody–associated disease (42) and anti-NMDAR encephalitis (43), we hypothesize that efgartigimod exerts its effects through dual mechanisms. First, by targeting FcRn, efgartigimod may limit the entry of peripheral IgG antibodies into the CNS while simultaneously enhancing their degradation. Second, in instances of BBB disruption, efgartigimod may gain partial access to the CNS, enabling its direct antagonism of pathogenic antibodies. Further investigations are warranted to validate these proposed mechanisms and fully elucidate the therapeutic potential of efgartigimod for AE.

The effects of efgartigimod observed in this study cannot be attributed exclusively to its use alone, as standard therapies may have had synergistic or additive effects, particularly given the duration of action of IVIG (44). Thus, we cannot definitively interpret the clinical courses of the reported cases as being due solely to the effect of efgartigimod. Additionally, the possibility of a placebo effect cannot be ruled out. Moreover, the cases reported here are from a single hospital; comprehensive investigations and experimental studies conducted with larger patient cohorts are needed to elucidate the mechanisms underlying the observed effects. Lastly, longitudinal multicenter studies are needed to determine the optimal efgartigimod treatment frequency and dosage and to clarify the drug’s long-term safety and efficacy. Notwithstanding these considerations, the findings from these cases offer valuable insights into potential treatment strategies for IgG-associated AE, and we will maintain ongoing follow-up of these patients.

## Conclusion

The symptoms of AE are diverse, with each subtype presenting distinct clinical manifestations. The delay of immunotherapy can lead to prolonged disease progression and the increased occurrence of residual sequelae, adversely affecting patient survival. Current first- and second-line treatments have inherent limitations, and efgartigimod has been demonstrated to be rapid and effective in the treatment of AE, showing promise for the reduction of corticosteroid dosages. It is a potential treatment option for AE.

## Data Availability

The original contributions presented in the study are included in the article/**Supplementary Material**. Further inquiries can be directed to the corresponding authors.
