# Supplementary material for: Case report: Rapid symptom relief in autoimmune encephalitis with efgartigimod: a three-patient case series
Source: Front Immunol. 2024 Oct 3;15:1444288. doi: 10.3389/fimmu.2024.1444288 (PMC11484013; doi:10.3389/fimmu.2024.1444288)
Supplement: Supplementary file 1 [file DataSheet1.docx]

**Supplementary Materials**


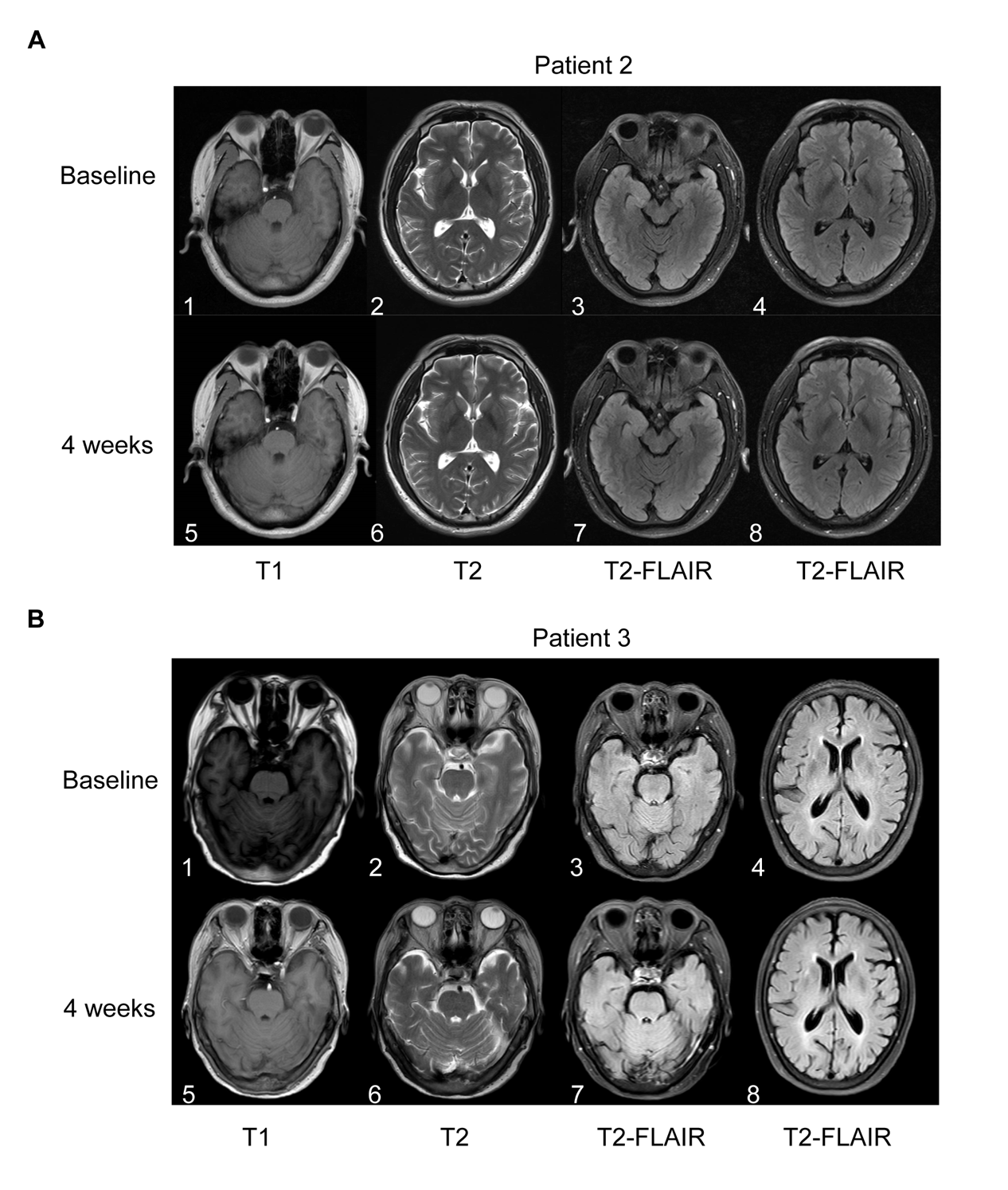


**Supplementary Figure 1.** Brain imaging results for patients 2 (A) and 3 (B) at baseline and after 4 weeks of efgartigimod treatment. FLAIR, fluid attenuated inversion recovery.
